# Physical activity in patients with systemic sclerosis

**DOI:** 10.1007/s00296-017-3879-y

**Published:** 2017-11-18

**Authors:** S. I. E. Liem, J. M. T. A. Meessen, R. Wolterbeek, N. Ajmone Marsan, M. K. Ninaber, T. P. M. Vliet Vlieland, J. K. de Vries-Bouwstra

**Affiliations:** 1 Department of Rheumatology, Leiden University Medical Centre, C1-51, PO Box 9600, 2300 RC Leiden, The Netherlands; 2 Department of Orthopedics, Leiden University Medical Centre, Leiden, The Netherlands; 3 Department of Medical Statistics, Leiden University Medical Centre, Leiden, The Netherlands; 4 Department of Cardiology, Leiden University Medical Centre, Leiden, The Netherlands; 5 Department of Pulmonology, Leiden University Medical Centre, Leiden, The Netherlands

**Keywords:** Systemic sclerosis, Scleroderma, Physical activity, Activities of daily living, Exercise

## Abstract

**Objectives:**

To compare self-reported levels of physical activity (PA) of systemic sclerosis (SSc) patients with the general population. To evaluate in SSc patients factors associated with PA levels and needs and preferences regarding PA.

**Methods:**

Fifty nine SSc patients completed the Short QUestionnaire to ASsess Health-Enhancing PA. The proportion of patients meeting the Dutch Recommendation for PA (= moderate PA for 30 min on ≥ 5 days/week) and total minutes of PA per week were calculated and compared with similar data from the Dutch population. Characteristics were univariately and multivariately compared between patients with low and high PA levels (either ≤ or > mean minutes/week of the Dutch population). Needs and preferences regarding PA promotion and guidance related to exercise were assessed by questionnaires.

**Results:**

Stratified for age (< 55 or ≥ 55 years) and gender, the proportion SSc patients meeting the Dutch recommendation for PA was not significantly different from the Dutch population. The total minutes of PA per week was significantly lower among SSc patients (1704 vs. 2614, *P* < 0.001). Multivariable analyses showed that in SSc patients the male gender, scleroderma health assessment questionnaire (SHAQ) and lack of energy were significantly associated with lower PA levels (*P* = 0.007; *P* = 0.042; *P* = 0.025). Two-third of patients required more information about PA.

**Conclusion:**

In SSc patients, the total minutes of PA per week are significantly lower compared to the general population. The male gender, functional ability as reflected by SHAQ and lack of energy seem to interfere with PA. These results might guide health professionals in providing their patients with appropriate information on PA.

**Electronic supplementary material:**

The online version of this article (10.1007/s00296-017-3879-y) contains supplementary material, which is available to authorized users.

## Introduction

Systemic sclerosis (SSc) is a rare autoimmune disease that is characterised by fibrosis and vasculopathy and clinically by Raynauds phenomenon and involvement of multiple organ systems, such as the gastrointestinal tract, kidneys, heart, lungs and musculoskeletal system. Based on the extent and localisation of skin involvement SSc, is subdivided into diffuse cutaneous SSc and limited SSc [3].

As a result of the impact of SSc on patients’ overall health status, the exercise capacity of SSc patients was found to be limited as well [4]. Studies on exercise capacity in SSc patients have so far mainly focused on aerobic capacity, and included maximal and submaximal endurance tests [5, 6]. However, from the patient’s perspective physical activity (PA) incorporated in a person’s daily life may be even more relevant. PA should be distinguished from exercise although the terms are often used interchangeably. PA includes exercise as well as other activities which involve bodily movement and are done as part of playing, working, active transportation, house chores and recreational activities. Exercise is a subcategory of physical activity that is planned, structured, repetitive, and purposeful in the sense that the improvement or maintenance of one or more components of physical fitness is the objective [7].

Whereas in other rheumatic conditions [8], including rheumatoid arthritis [9], osteoarthritis [10], spondyloarthritis [11, 12] and systemic lupus erythematosus [13], levels of PA were found to be lower than those of the general population, research in SSc is scarce. A recent study concluded that approximately half of the SSc patients engaged in exercise and that these patients exercised on average 4.7 h per week [14]. In addition, another study found that although SSc patients reported an overall lower capacity for walking, jogging and running and more limiting factors for physical capacity than population-based controls, there were no differences in reported PA and time sitting [15]. Moreover, daily physical activities can also be assessed by more objective instruments such as a portable multiple sensor device. This study concluded that daily physical activities were significantly reduced in 27 SSc patients as compared to 11 controls patients [15, 16]. However, to our knowledge no study has assessed the overall level of PA in minutes per week and adherence to guidelines of PA for patients with SSc and compared this to the general population. This information is relevant, as it is generally acknowledged that for patients with rheumatic conditions, regular PA does not only have general health benefits but has a beneficial impact on disease-related consequences such as pain, stiffness, and fatigue as well [17].

Together with the evaluation of SSc patient’s preferences regarding guidance to obtain and maintain a sufficient level of PA, knowledge on PA levels can stimulate specific guidance of SSc patients in improving a healthy lifestyle including sufficient levels of PA and appropriate exercise related to their condition.

Therefore, the current study aimed to determine the level of PA in patients with SSc as well as factors associated with PA. We evaluated the PA levels in patients with SSc and compared these to the levels of the general Dutch population. In addition, we evaluated whether specific disease characteristics are associated with the PA levels in SSc patients and specific needs and preferences regarding PA promotion and guidance related to exercise among SSc patients.

## Patients and methods

### Study design

This analysis is part of a large prospective cohort study on patients with SSc participating in an annual comprehensive care program [18]. This annual care program started in 2009 (and is still operational now, in October 2017) and all patients participating in this program provided written informed consent for using gathered data for clinical analyses. This standardised 2-day care pathway comprises a visit to the rheumatologist, pulmonologist and cardiologist. In addition to the extensive medical screening, patients are routinely seen by a physical therapist for several measurements and advice regarding PA and exercise and/or referral to a first line physical therapist. For every patient, the care pathway is performed on 2 consecutive days between 8:00 and approximately 16:00. All patients, in whom a diagnosis of SSc is confirmed during the first visit to the care pathway, are included for the annual follow-up. The present study was performed in a group of 59 patients who visited an information meeting on SSc and the care program (September 2014). All patients attending the meeting were asked to fill in questionnaires specifically concerning levels of PA and needs for instructions to exercise. Additional data concerning clinical characteristics and functional ability were gathered in the context of the prospective follow-up study.

A comparison of patients’ PA levels with those of the general population was made using aggregated, publicly available data acquired from a health survey of the Central Bureau for Statistics (CBS; http://statline.cbs.nl/Statweb/).

### Patients

Admission criteria for the care program included a diagnosis of SSc according to the referring rheumatologist, or a strong suspicion for SSc and a request for a complete diagnostic work-up to confirm the diagnosis. Patients can be referred by rheumatologists from the outpatient clinic of the LUMC or from any other hospital in The Netherlands [18]. All included SSc patients fulfilled the criteria of the American College of Rheumatology 1980 or LeRoy Criteria for SSc and 51 (88%) fulfilled the American College of Rheumatology 2013 criteria for SSc [19, 20].

### General population

The data from the CBS are gathered annually by sending health questionnaires to a sample of the Dutch population. These surveys include measures of PA and quality of life. The Short QUestionnaire to ASsess Health-Enhancing PA (SQUASH) was used to measure levels of PA. Aggregated data, categorized by sex and age (categories: ≥20 years, 20–55 years, ≥ 55 years) were obtained from the CBS (Appendix 1 in Electronic supplementary materials). For all subtotals of the SQUASH and the total SQUASH, mean values, standard errors of the mean, standard deviations and the number of respondents were provided, allowing statistical comparisons with the data from the SSc patients. For the present analysis, data of 2013 were used.

### Main outcome variables

#### Physical activity

PA was measured using the validated Dutch version of the Short QUestionnaire to Assess Health-Enhancing PA (SQUASH) [21]. The SQUASH consists of 17 items asking respondents to recall PA as performed during a regular week, categorized into commuting activities, activities at work and school, walking, cycling, household activities, gardening, odd jobs, leisure time activities and sport activities. Patients wrote down how many days per week and how many hours per day they spent performing these activities, which made it possible to calculate the total minutes per week spent on PA. With the aid of the Ainsworth compendium assigning the metabolic equivalents (METS) to each activity [22], we then could define the intensity of the PA. This information was used to define if an individual adhered to the Dutch Recommendation for Health-Enhancing PA.

The following definition of the Dutch Recommendation for Health-Enhancing PA for adults aged between 18 and 55 years was used: “at least 30 min of PA with moderate intensity (≥ 4 METs) on more than 5 days per week”. For adults aged 55 and above the Dutch Recommendation for Health-Enhancing PA is defined as “at least 30 min of PA with moderate intensity (≥ 3 METs) on more than 5 days per week” [23].

#### Needs, preferences and perceptions regarding PA promotion and guidance related to exercise

The patients’ perceptions on PA and their need for information were assessed using 26 statements (Appendix 2 in Electronic supplementary materials). Three statements concerned the need for more information regarding PA and exercise and four statements for the need for instructions to exercise. The other 19 statements assessed the opinions and knowledge of patients with SSc about PA promotion and guidance related to exercise. Given considerable overlap between part of the statements, data were summarized according to the subject addressed. Complete data of each individual statement are shown in Appendix 2 in Electronic supplementary materials, Table 1.

**Table 1:** Characteristics of participating systemic sclerosis patients (*N* = 59)

Sociodemographic characteristics	
Age, years, median (interquartile range)	65 (55–70)
Female, *N* (%)	52 (88%)
Caucasian origin, *N* (%)	48 (81%)
Smoking	
Currently, *N* (%)	3 (5%)
Past, *N* (%)	29 (49%)
Body mass index, kg/m^2^, mean (SD)^a^	25 (4)
Disease characteristics	
Type of systemic sclerosis, *diffuse, N* (%)	15 (25%)
Duration of Raynaud’s phenomenon, years, median (interquartile range)	12 (6–22)
Duration of non-Raynaud’s phenomenon, years, median (interquartile range)	6 (3–16)
Disease duration, years, median (interquartile range)	4 (2–15)
Modified Rodnan Skin Score, median (interquartile range)	3 (0–6)
Proximal muscular weakness or synovitis, *N* (%)	6 (10%)
Joint contractures or atrophy, *N* (%)	11 (19%)
Gastrointestinal involvement, *N* (%)^b^	41 (76%)
Immunotherapy, current or past, *N* (%)	38 (64%)
Anti-Scl-70, *N* (%)	9 (15%)
Anti-centromere, *N* (%)	29 (49%)
RNA polymerase III, *N* (%)	3 (5%)
6 min walking distance: total distance, mean (SD)	511 (84)
Decrease in DLCO% of predicted, *N* (%)^a^	35 (60%)
Interstitial lung disease according HRCT, *N* (%)	26 (45%)
Pulmonary arterial hypertension, *N* (%)	6 (11%)
Decreased ejection fraction, *N* (%)	7 (13%)
Arrhythmia	24 (46%)
Scleroderma health assessment questionnaire, median (interquartile range)	0.75 (0.25–1.125)

^a^DLCO: diffuse capacity for carbon monoxide. A reduced DLCO was defined as < 70

^b^Gastrointestinal involvement was defined as the presence of one of the following symptoms: reflux, early satiety, vomiting, diarrhoea, intestinal distension, constipation, faecal incontinence, parenteral nutrition or dysphagia

The statements were graded on a five point Likert scale, ranging from totally agree to totally disagree. For analyses, the scores were dichotomized in two categories: 0: “totally disagree, disagree, indifferent” and 1: “agree or totally agree”.

### Assessments

Of all patients, sociodemographic data, disease characteristics and a measure of daily functioning were obtained from the database of the larger cohort study. For the current study, data gathered during the visit to the care program closest to the date of the information meeting were used.

Sociodemographic characteristics included age, gender, origin, smoking habits and Body Mass Index (BMI, kg/m^2^). Furthermore, we checked for significant comorbidity interfering with PA and exercise and not related to SSc by manually reviewing the medical records. Significant comorbidity included osteoarthritis resulting in joint prosthesis, limb amputation and cardiovascular or pulmonary comorbidity.

Disease characteristics included type of SSc (diffuse or limited), duration of Raynaud’s phenomenon, duration of non-Raynaud’s phenomenon (time since first symptom other than Raynaud’s phenomenon) and disease duration (time since diagnosis SSc was confirmed by a physician). The extent and severity of skin involvement was measured by means of the modified Rodnan Skin Score (mRSS) and ranged from 0 [normal] to 3 [most severe] on 17 different body parts combining to a total maximum score of 51 [24].

Active joint/muscle involvement was defined as the presence of proximal muscular weakness, synovitis or both. Muscle strength was assessed by an experienced rheumatologist and graded on a 0–5 scale, patients with a score < 5 were classified as having proximal muscle weakness. The presence of synovitis was assessed by an experienced rheumatologist [25]. The presence of joint contractures or atrophy was also included (yes when either joint contractures, atrophy or both were present). Gastrointestinal involvement was defined as the presence of at least one of the following symptoms: reflux, early satiety, vomiting, diarrhoea, intestinal distension, constipation, faecal incontinence, parenteral nutrition or dysphagia.

The use of immunosuppressive therapy (yes/no), presence of anti-Scl70 antibodies (yes/no), anti-centromere antibodies (yes/no) and presence of RNA polymerase III antibodies were collected as well.

Additionally, a 6 min walking test (total distance in meters) was performed. Lung functioning was assessed by measuring the diffusing capacity for carbon monoxide (% of predicted; a reduced diffusing capacity for carbon monoxide was defined as < 70 [26]). Diagnosis of interstitial lung disease (ILD; yes/no) was determined based on the presence of interstitial fibrosis or ground glass opacities on high-resolution computerised tomography as reported by the radiologist.

The systolic pulmonary artery pressure and left ventricular ejection fraction were estimated using echocardiography by an experienced cardiologist. Elevated pulmonary pressure was defined using a cut-off value of 35 mm Hg [27]. A decreased ejection fraction was defined as ≤ 54% [28]. Presence of arrhythmias was defined as presence of multiform ventricular extrasystoles > 100/day, couplets or runs of ventricular tachycardia or supraventricular tachycardia or at least 30 s on 24 h Holter monitoring.

Patients were asked to fill in the Scleroderma Health Assessment Questionnaire (SHAQ) to measure daily functioning. This is a 20-item questionnaire comprising eight domains of activities of daily living, with the final score ranging from 0 (no disability) to 3 (severe disability) [29].

### Statistical analysis

For the patients’ sociodemographic and disease characteristics descriptive statistics were used.

According to their distribution, continuous variables were either presented as mean and standard deviation (SD) or medians with interquartile range (p25–p75). Categorical variables were presented as frequencies with percentages.

To compare the characteristics of patients participating in the present analysis with the other patients taking part in the annual care program, Mann–Whitney *U* or Chi-square tests were used. The following characteristics were compared between the included patients in this study and the rest of the cohort: age, gender, BMI, type of SSc, duration of Raynaud’s phenomenon, duration of non-Raynaud’s phenomenon, disease duration, modified Rodnan skin score, proximal muscular weakness or synovitis, gastrointestinal involvement, anti-Scl-70 antibodies, anti-centromere antibodies, RNA polymerase III antibodies, interstitial lung disease, pulmonary arterial hypertension, decreased ejection fraction and arrhythmia.

Comparisons of proportion of persons fulfilling the Dutch Recommendation for Health-Enhancing PA to the proportion of the Dutch population were performed with a logistic regression.

Comparisons of the total minutes per week spent on PA between SSc patients and the Dutch population were done by a *t* test. Data of the Dutch population were aggregated which was taken into account when using *t* tests.

Furthermore, percentages of SSc patients agreeing with the different items exploring needs, preferences and perceptions for the delivery of PA promotion and guidance related to exercise were computed.

Finally, univariate analyses of variance were performed for each characteristic to determine whether characteristics influenced the total minutes of PA. This was done for the following characteristics: age, gender, BMI, type of SSc, duration of Raynaud’s phenomenon, duration of non-Raynaud’s phenomenon, disease duration, modified Rodnan skin score, proximal muscular weakness or synovitis, atrophy or joint contractures, gastrointestinal involvement, 6 minute walking distance, reduced diffuse capacity for carbon monoxide, interstitial lung disease, pulmonary arterial hypertension, decreased ejection fraction, arrhythmia, the SHAQ, pain during exercise and lack of energy. Then, characteristics which were univariately associated with the total minutes of PA (*p* value of < 0.2) were entered in a stepwise multiple linear regression model.

Data entry was performed using Microsoft Office Access 2003. All statistical analyses were executed using SPSS 23.0 software (SPSS Inc., Chicago, USA).

## Results

Seventy-nine patients attended an information meeting and 59 (75%) completed the questionnaire on PA. The median duration between the information meeting and the date of clinical evaluation in the health care program was 145 days (interquartile range 76–387).

### Participants

Table 1 shows the characteristics of the 59 participating SSc patients. These included 52 women and 7 men, ranging in age from 20 to 84 years (median age 65 years) with disease duration ranging from 1 month to 38 years (median 4 years). 14 (24%) SSc patients were younger than 55 years. 15 (25%) patients had diffuse SSc and 44 (75%) limited cutaneous SSc. One patient used a rollator because of walking problems caused by peripheral vasculopathy and thus had comorbidity not directly related to SSc and possibly interfering with PA.

In September 2014, the cohort of SSc patients consisted of 303 participants. A comparison between the included SSc patients (*N* = 59) in this study and the rest of the cohort (*N* = 244) showed that the included SSc patients were significantly older than the rest of the cohort (65 vs. 54 years, *P* < 0.001). Additionally, the proportion of included SSc patients in this study positive for anti-centromere antibodies was higher than the proportion of the rest of the cohort (51 vs. 32%, *P* = 0.006). No other variable was significantly different between both groups (Appendix 3 in Electronic supplementary materials).

### Proportion of persons with SSc and the general population meeting the Dutch Recommendation for Health-Enhancing PA

The proportions of SSc patients meeting the Dutch Recommendation for Health-Enhancing PA are reported in Table 2. There was no significant difference in proportion of SSc patients meeting the Dutch Recommendation for Health-Enhancing PA as compared to the Dutch population (68 vs. 61%; *P* = 0.951).

**Table 2:** Proportions of patients and persons from the general population meeting Dutch Recommendation for Health-Enhancing Physical Activity (%)

	Patients with SSc (*N* = 59)	Dutch population (*N* = 5789)	Odds ratio Dutch population vs. SSc (95% confidence interval)	*p* value
Total				
< 55	57% (*N* = 8)	51% (*N* = 1609/3142)	0.787 (0.273–2.274)	0.658
≥ 55	71% (*N* = 32)	72% (*N* = 1919/2647)	1.071 (0.559–2.052)	0.836
All	68% (*N* = 40)	61% (*N* = 3528/5789)	0.983 (0.561–1.721)	0.951
Women				
< 55	57% (*N* = 8)	51% (*N* = 909/1769)	0.793 (0.274–2.294)	0.668
≥ 55	71% (*N* = 27)	69% (*N* = 920/1333)	0.908 (0.446–1.847)	0.789
All	67% (*N* = 35)	59% (*N* = 1829/3102)	0.870 (0.481–1.575)	0.645
Men				
< 55	–	51% (*N* = 700/1373)	NA	NA
≥ 55	71% (*N* = 5)	76% (*N* = 999/1314)	1.269 (0.245–6.6571)	0.777
All	71% (*N* = 5)	63% (*N* = 1699/2687)	NA	NA

The comparisons between the patients with systemic sclerosis and the Dutch population were done with a logistic regression relating group (systemic sclerosis or Dutch population) to adherence of the Dutch Recommendation for Health-Enhancing Physical Activity (yes/no)

*NA* not applicable, *SSc* systemic sclerosis

Subgroup analyses according to age and gender showed that the proportion of male SSc patients fulfilling the Dutch Recommendation for Health-Enhancing PA was lower than the male Dutch population (71 vs. 76%; *P* = 0.777). Both the proportions of female SSc patients aged younger than 55 years and aged 55 years older and fulfilling the recommendations for PA were higher than the Dutch population (respectively 57 vs. 51%, *P* = 0.668 and 71 vs. 69%, *P* = 0.789). Stratification for BMI (BMI < 25 vs. BMI ≥ 25) did not show a difference in the proportion of patients fulfilling the Dutch Recommendation for Health-Enhancing PA.

Excluding the SSc patient with significant comorbidity other than SSc and possibly interfering with PA, these results did not change.

### Time spent on PA in patients with SSc and the Dutch population

Table 3 describes the minutes per week spent on PA in patients with SSc and the general population. Regarding the total minutes spent on PA, SSc patients were significantly less physically active than the Dutch population (1704 vs. 2614 min/week, *P* < 0.001; relative difference 35%).

**Table 3:** Minutes per week spent on physical activity

	Total	Women		Men
	(*N* = 59)^a^	< 55	≥ 55	≥ 55
		(*N =* 14)^a^	(*N* = 38)^a^	(*N* = 7)^a^
Total activities (min/week), mean (SD)				
SSc	1704 (1013)	1944 (1176)	1778 (939)	818 (637)
DP	2614 (1422)	2951 (1280)	2015 (1423)	2065 (1460)
* p* value*	< *0.001*	*0.001*	0.131	< *0.001*
Working or school activities (min/week), mean (SD)				
SSc	363 (711)	827 (931)	258 (608)	1 (4)
DP	1162 (1097)	1312 (919)	376 (720)	672 (1023)
* p* value*	< *0.001*	0.052	< *0.001*	< *0.001*
Commuting (min/week), mean (SD)				
SSc	48 (132)	57 (78)	54 (157)	0 (0)
DP	93 (257)	123 (258)	47 (198)	76 (278)
* p* value	*0.010*	*0.002*	0.788	< *0.001*
Household activities (min/week), mean (SD)				
SSc	785 (609)	620 (508)	945 (596)	244 (516)
DP	745 (799)	1027 (928)	1012 (858)	474 (592)
* p* value*	0.617	*0.003*	0.501	0.240
Leisure time activities (min/week), mean (SD)				
SSc	507 (425)	440 (355)	534 (464)	572 (345)
DP	617 (639)	520 (466)	440 (355)	848 (812)
* p* value*	*0.049*	0.579	0.367	*0.037*
Sport (min/week), mean (SD)				
SSc	87 (115)	108 (74)	88 (135)	56 (50)
DP	141 (257)	86 (134)	108 (74)	131 (294)
* p* value*	< *0.001*	0.460	0.858	< *0.001*

All *p* values < 0.05 are considered significant and shown in italics

*SSc* systemic sclerosis patients, *DP* Dutch population

* The analysis to compare the amount of minutes per week spent on physical activity between the patients with systemic sclerosis and the Dutch population was done with a *t* test

^a^Numbers represent the number of SSc patients in each group

Male SSc patients aged 55 years and older, as well as female SSc patients aged 55 years and younger, spent significantly less minutes on PA per week than their male and female controls from the Dutch population (respectively 818 vs. 2065 min/week with *P* < 0.001 and 1944 vs. 2951 min/week with *P* = 0.001).

When splitting the total PA into different categories, SSc patients generally spent less minutes per week on the majority of different activities than the Dutch population, except household activities in the total group (785 vs. 745 min/week, *P* = 0.492.

Stratified for gender, female SSc patients aged 55 years and younger spent significantly less minutes per week than the female Dutch population on total activities (1944 vs. 2951 min/week, *P* = 0.001), commuting (57 vs. 123 min/week, *P* = 0.002) and household activities (620 vs. 1027 min/week, *P* = 0.003). The female SSc patients aged 55 years and older spent significantly less minutes on working or school activities (258 vs. 376 min/week, *P* < 0.001). The male SSc patients aged 55 years and older spent significantly less minutes per week than the male Dutch population on total activities (818 vs. 2065 min/week, *P* < 0.001), working or school activities (1 vs. 672 min/week, *P* < 0.001), commuting (0 vs. 76 min/week, *P* < 0.001), leisure time activities (572 vs. 848 min/week, *P* = 0.037) and sporting activities (56 vs. 121 min/week, *P* < 0.001).

Excluding the patient with severe comorbidity other than SSc and possibly interfering with PA, two categories of physical activities changed for SSc patients. First, excluding this patient, SSc patients performed 514 min per week on leisure time activities; 7 min less without excluding this SSc patient. Excluding this patient, the comparison of leisure time activities between the SSc patients and the Dutch population is no longer significant (*P* = 0.070). Second, excluding this SSc patient with comorbidity, female SSc patients aged 55 years and older performed 265 min per week on working or school activities; 7 min more than without excluding this patient. Excluding this SSc patient, the comparison of working or school activities between the female SSc patients aged 55 years and older and the female Dutch population aged 55 years and older is no longer significant (*P* = 0.281).

### SSc patients’ needs, preferences and perceptions regarding PA promotion and guidance related to exercise

Table 4 shows the needs, preferences and perceptions regarding PA promotion and guidance related to exercise of the SSc patients. Thirty seven (66%) patients were satisfied with their current physical activities, whereas 19 (34%) patients wanted to be more physically active. Fourteen (22%) SSc patients wished to have additional instructions related to PA and exercise. Of those 13 patients, eight patients wanted to receive guidance on a weekly basis and two patients according to their need. Furthermore, nine of those 13 patients who wanted additional instructions related to PA and exercise, wished to receive individual instructions for general or specific physical therapy (*N* = 1 and *N* = 3) or both (*N* = 5), and six patients wished to have instructions in groups. Concerning group instructions, one patient wanted to have these at the gym, one together with other SSc patients, one with patients with a rheumatic disease and two patients a combination of these previous options.

**Table 4:** Perceptions of systemic sclerosis patients regarding physical activity promotion and guidance related to exercise

	Agreed or highly agreed, *N* (%)
I am satisfied with my current level of physical activities	37 (66%)
I need guidance to PA and/or exercise	13 (23%)
I would like to have guidance once a week^a^	8/13 (62%)
I would like to have guidance when I need it^a^	2/13 (15%)
I would like to have guidance individually^a^	9/13 (69%)
I would like to have guidance in groups^a^	6/13 (46%)
I need more information about physical activity and sports, physical therapy, or physical therapists with specific knowledge and skills regarding the management of systemic sclerosis	39 (68%)
Patients with systemic sclerosis	
Have their own responsibility to be sufficiently physically active	54 (92%)
Rely on advice of their rheumatologists and general practitioners regarding PA and exercise	17 (29%)
The type of PA (including exercise and sports) I would prefer	
Is not appropriate for people with a disease such as systemic sclerosis	10 (19%)
Could damage my joints, lungs, heart or skin	9 (16%)
After being physically I get a lot of pain	18 (32%)
In generally, I do not	
Have enough energy to engage in exercise or sports	16 (27%)
Have enough time to engage in exercise play sports	5 (9%)
Want to engage in exercise or play sports	10 (17%)
Sufficient levels of physical activity have a beneficial effect on the health status of persons with systemic sclerosis	48 (81%)

Missing numbers not listed

Answers were collected on a Likert scale and then transformed in dichotomous outcomes (agree or highly agree = 1, highly disagree, disagree or indifferent = 0)

^a^Patients could choose multiple options

Forty-three (80%) SSc patients indicated that they thought PA can have beneficial effects on their health status and that being physically active is not harmful. Nevertheless, 39 (66%) SSc patients stated that they needed additional information about exercise and sports, occupational therapy and physical therapy. One-third of the SSc patients reported to rely on advice of their rheumatologist and general practitioner with regard to advice on PA and exercise.

Furthermore, 18 SSc patients (32%) stated to suffer from a lot of pain due to PA including sports. SSc patients who did not meet the Dutch Recommendation for Health-Enhancing PA more often reported to suffer from pain due to PA compared to SSc patients who did meet the Dutch Recommendation for Health-Enhancing PA (9/18 [50%] vs. 9/38 [24%], *P* = 0.049). Sixteen (27%) SSc patients stated that lack of energy interfered with being active. SSc patients who did not meet the Dutch Recommendation for Health-Enhancing PA more often reported a lack of energy interfering with PA than SSc patients who did meet the Dutch Recommendation for Health-Enhancing PA (10/19 [53%] vs. 6/40 [15%], *P* = 0.002).

### Disease characteristics associated with PA levels in SSc patients

As shown in Table 5, univariate analyses of patient characteristics demonstrated that age, gender, proximal muscular weakness and synovitis, interstitial lung disease, the SHAQ, pain during exercising and a lack of energy were associated with total minutes of PA per week (*P* < 0.2). For example, a male SSc patient spent 1005 min per week less on PA than female SSc patients. Multivariable analysis showed that male gender, the SHAQ and a lack of energy were the factors significantly associated with the amount of minutes spent on PA in the SSc group (respectively *P* = 0.007, *P* = 0.042 and *P* = 0.025).

**Table 5:** Association between disease characteristics and number of minutes of physical activity

Variables in analysis	Univariate analyses^a^		Multivariate analysis^b^	
	Coefficient	95% confidence interval	Coefficient	95% confidence interval
Age	− 15	(− 36, 7)		
Male gender	− 1005	(− 1785, − 225)	− 1025	(− 1751, − 298)
Proximal muscular weakness or synovitis	− 585	(− 1462, 291)		
Interstitial lung disease	− 645	(− 1162, 128)		
Scleroderma Health Assessment Questionnaire	− 492	(− 892, − 93)	− 444	(− 830, − 58)
Pain during exercise	− 595	(− 1167, − 22)		
Lack of energy	− 860	(− 1415, − 306)	− 571	(− 1121, − 22)

Shown are beta for “yes” of reference category for all categorical variables

^a^Univariate linear regressions were done relating minutes per week spent on physical activity to individual variables

^b^Multivariate linear regression analysis included all variables with a *P* value < 0.2 in the univariate analyses. Only significant outcomes were reported

Excluding the SSc patient with significant comorbidity other than SSc and possibly interfering with PA, these results did not change.

## Discussion

To the best of our knowledge, this is the first study that evaluated levels of physical activity (PA) in SSc patients and compared these levels to the general Dutch population. Our data show that, stratified for age and gender, the proportions of SSc patients meeting the Dutch Recommendation for Health-Enhancing PA was not significantly different from the Dutch population. However, with respect to the mean number of minutes of PA per week, the SSc patients were less physically active. In patients not meeting the Dutch Recommendation for Health-Enhancing PA pain and lack of energy might interfere with PA. Multivariable analysis showed that the male gender, lower functional ability as reflected by the SHAQ and a lack of energy were associated with lower levels of PA. As PA has been shown to be beneficial, these results underline the importance of proper guidance of SSc patients regarding a healthy lifestyle including appropriate levels of PA. In addition, factors causing pain and lack of energy should be addressed, as they seem to interfere with attaining and maintaining sufficient levels of activity. The difference between the proportion of patients meeting the Dutch Recommendation for Health-Enhancing PA on the one hand, and the mean number of minutes PA per week on the other hand, shows that solely meeting a recommendation is not a reliable way to asses PA. No previous study has assessed self-reported levels of PA for SSc patients. However, our results are in line with previous research in patients with rheumatoid arthritis (RA), where the proportion of RA patients meeting the Dutch Recommendation for Health-Enhancing PA was comparable to the general population, but the mean number of minutes per week spent on PA was lower [9]. Strikingly, SSc patients in the current study performed more minutes per week on PA than the RA patients (1704 vs. 1535 min/week) in the previous study [9]. This could possibly be explained by the specific information and physical therapy that the SSc patients in the current study receive as part of their care program. Another reason might be that RA is characterised by synovitis and joint complaints, while in SSc specific joint problems are less frequent (10% of the included SSc patients had proximal muscular weakness or synovitis). However, data on joint involvement in the RA study are lacking. Another explanation could be that the study in RA patients was performed over 10 years ago. Currently, the proportion of persons meeting the public health recommendation for healthy PA has increased in general in the Netherlands [30].

Regarding the relevance of our findings, it is plausible that, like in patients with other rheumatic conditions, enhancing PA levels according to levels recommended for the general population, including elderly and patients with chronic diseases [31], may improve physical functioning or prevent deterioration in SSc patients. Beneficial effects of PA have been demonstrated in many different diseases such as diabetes mellitus type 2 and rheumatoid arthritis [32, 33]. Moreover, beneficial outcomes regarding aerobic capacity and muscle strength have been reported [5, 34, 35]. Indeed, with respect to safety of PA in patients with SSc, various studies have demonstrated that exercise programs with moderate to even vigorous intensity did not have detrimental effects. For example, a multidisciplinary team-care program of 12 weeks implying daily and structured exercise in a randomized controlled design, resulted in less limitations in activities, and improvement in mouth opening and hand grip strength. These improvements were still present after 24 weeks but no longer significant, suggesting a need for continuous guidance to encourage SSc patients to exercise [35]. These results can, however, not be extrapolated to the total population of patients with SSc, as they concern selected groups of patients, in whom contra-indications for vigorous exercise were ruled out. Given the potential risks of high intensity PA in this patient group, regular screening of the heart and the lungs by monitoring pulse oximetry and heart rate and supervision by health professionals during exercise, seems warranted.

Previous literature showed that involvement of the cardiopulmonary system can result in a reduced exercise tolerance [36–38]. Indeed, interstitial lung disease was associated with lower levels of PA in our study in the univariate analysis. No other characteristics reflecting severe organ involvement like left ventricular ejection fraction and diffuse cutaneous SSc, were associated with levels of PA in our study. Various explanations might account for this observation. The number of included patients is low, resulting in possible lack of power to find an association. For example, the wide confidence interval for the association between interstitial lung disease and lower levels of PA (− 1162 to 128) supports this explanation. Additionally, by including patients visiting an information meeting, it is possible that patients with the most severe disease activity did not participate. For example, only a small portion of patients had a decreased left ventricular ejection fraction (*N* = 7, 13%), and the proportion of anti-centromere positive patients was higher as compared to all patients participating in the care program (51 vs. 32%).

In this study, pain during exercise and a lack of energy were interfering with exercise. It is, therefore, important to determine the reasons behind these two factors because solving this might enhance PA. Furthermore, female SSc patients spent more time on PA than men. Conclusions should be drawn with caution, given the low percentage of men included. However, the percentage of men did not differ from the total cohort, justifying carefully monitoring PA in male patients.

Finally, given that nearly two-third of the SSc patients wanted to receive more information concerning PA promotion and guidance related to exercise and therapy and roughly one-third reported to rely on the advice of their rheumatologist or general practitioner, health care providers should be aware of these expectations among their patients.

This study has some limitations that should be taken into account. First, data were gathered by means of self-reporting questionnaires which could lead to bias. Second, the data of the Dutch population were only available on an aggregated level, limiting the possibility of using several statistical analyses.

In conclusion, in SSc patients, the total minutes of PA per week is significantly lower compared to the general population. The male gender, functional ability as reflected by SHAQ and lack of energy seem to interfere with PA. One-third of the SSc patients expressed the need to have more information concerning physical activity promotion and guidance related to exercise. These results might guide health professionals in providing their patients with appropriate information on physical activity.

## Electronic supplementary material

Below is the link to the electronic supplementary material.

Supplementary material 1 (DOCX 26 KB)

Supplementary material 2 (DOC 130 KB)

Supplementary material 3 (DOC 82 KB)
