# Supplementary material for: Physical activity in patients with systemic sclerosis
Source: Rheumatol Int. 2017 Nov 18;38(3):443–53. doi: 10.1007/s00296-017-3879-y (PMC5847038; doi:10.1007/s00296-017-3879-y)
Supplement: Supplementary file 1 — Supplementary material 1 (DOCX 26 KB) [file 296_2017_3879_MOESM1_ESM.docx]

**Physical Activity in Patients with Systemic Sclerosis**

SIE Liem, BSc^1^,

JMTA Meessen, MSc^2^,

R Wolterbeek, MSc^3^,

N Ajmone Marsan MD, PhD^4^,

MK Ninaber, MD, PhD^5^,

Prof. Dr. TPM Vliet Vlieland^2^,

JK de Vries-Bouwstra, MD, PhD^1^

^1^Department of Rheumatology, Leiden University Medical Centre, the Netherlands

^2^Department of Orthopedics, Leiden University Medical Centre, the Netherlands

^3^Department of Medical Statistics, Leiden University Medical Centre, the Netherlands

^4^Department of Cardiology, Leiden University Medical Centre, the Netherlands

^5^Department of Pulmonology, Leiden University Medical Centre, the Netherlands

**Corresponding author:**

J.K. de Vries-Bouwstra

Leiden University Medical Center, Department of Rheumatology, C1-51

PO Box 9600, 2300 RC Leiden, The Netherlands

Tel. [+31 71 526 3598](tel:+31%2071%20526%203598)

Fax.[+31 71 526 6752](tel:+31%2071%20526%206752)

E-mail [j.k.de_vries-bouwstra@lumc.nl](mailto:j.k.de_vries-bouwstra@lumc.nl)

**Appendix 1** – Data of the Dutch population

from the Central Bureau of Statistics
