# Supplementary material for: Physical activity in patients with systemic sclerosis
Source: Rheumatol Int. 2017 Nov 18;38(3):443–53. doi: 10.1007/s00296-017-3879-y (PMC5847038; doi:10.1007/s00296-017-3879-y)
Supplement: Supplementary file 2 — Supplementary material 2 (DOC 130 KB) [file 296_2017_3879_MOESM2_ESM.doc]

**Physical Activity in Patients with Systemic Sclerosis**

**Appendix 2 “The needs, preferences and perceptions regarding physical activity promotion and guidance related to exercise of systemic sclerosis patients”**

This appendix shows the format of the questionnaire used to assess the needs, preferences and perceptions regarding physical activity of patients with systemic sclerosis and the complete data of each individual statement.

______________________________________________________________________________

Explanation: below are several statements about your (patients with systemic sclerosis) needs and preferences regarding physical activity. Please indicate for all statements to which extent you agree.

|  | Totally disagree | Disagree | | Indifferent | | Agree | Totally agree |
| --- | --- | --- | --- | --- | --- | --- | --- |
| I (patient with systemic sclerosis) need more information about: |  | |  | |  |  |  |
| - Physical activity and sports | ❑ | | ❑ | | ❑ | ❑ | ❑ |
| - Physical therapy | ❑ | | ❑ | | ❑ | ❑ | ❑ |
| - Physical therapists with specific knowledge and skills regarding the management of systemic sclerosis | ❑ | | ❑ | | ❑ | ❑ | ❑ |
|  |  | |  | |  |  |  |
| Patients with systemic sclerosis |  | |  | |  |  |  |
| - Have their own responsibility to be sufficient physically active | ❑ | | ❑ | | ❑ | ❑ | ❑ |
| - Rely on advice of their rheumatologists and general practitioners regarding PA and exercise | ❑ | | ❑ | | ❑ | ❑ | ❑ |
| - Can only engage in exercise or sports if they are accompanied | ❑ | | ❑ | | ❑ | ❑ | ❑ |
| - Should get more help with making a choice for exercise or sports | ❑ | | ❑ | | ❑ | ❑ | ❑ |
|  |  | |  | |  |  |  |
| The type of PA (including exercise and sports) I prefer |  | |  | |  |  |  |
| - Is not available in my neighbourhood | ❑ | | ❑ | | ❑ | ❑ | ❑ |
| - Is not appropriate for people with a disease such as systemic sclerosis | ❑ | | ❑ | | ❑ | ❑ | ❑ |
|  |  | |  | |  |  |  |
| After being physically active I get a lot of pain. | ❑ | | ❑ | | ❑ | ❑ | ❑ |
|  |  | |  | |  |  |  |
| I have the feeling that I |  | |  | |  |  |  |
| - Damage my joints too much by engaging in exercise or sports | ❑ | | ❑ | | ❑ | ❑ | ❑ |
| - Damage my lungs too much by engaging in exercise or sports | ❑ | | ❑ | | ❑ | ❑ | ❑ |
| - Damage my heart too much by engaging in exercise or sports | ❑ | | ❑ | | ❑ | ❑ | ❑ |
| - Damage my skin too much by engaging in exercise or sports | ❑ | | ❑ | | ❑ | ❑ | ❑ |
|  |  | |  | |  |  |  |
| When participating with organized exercise activities for healthy persons I have the feeling of being watched by the other people because I have systemic sclerosis. | ❑ | | ❑ | | ❑ | ❑ | ❑ |
|  |  | |  | |  |  |  |
| In generally, I do not |  | |  | |  |  |  |
| - Have enough energy to engage in exercise or sports | ❑ | | ❑ | | ❑ | ❑ | ❑ |
| - Have enough time to engage in exercise or sports | ❑ | | ❑ | | ❑ | ❑ | ❑ |
| - Want to engage in exercise play sports | ❑ | | ❑ | | ❑ | ❑ | ❑ |
|  |  | |  | |  |  |  |
| In my neighbourhood there is no professional support for engaging in exercise or sports | ❑ | | ❑ | | ❑ | ❑ | ❑ |
|  |  | |  | |  |  |  |
| I am not well enough informed about which possibilities for PA are available for systemic sclerosis patients. | ❑ | | ❑ | | ❑ | ❑ | ❑ |
|  |  | |  | |  |  |  |
| Sufficient levels of physical activity have a beneficial effect on the health status of persons with systemic sclerosis. | ❑ | | ❑ | | ❑ | ❑ | ❑ |
|  |  | |  | |  |  |  |
| Daily activities, such as walking, for patients with systemic sclerosis could be improved by a better condition and more muscle strength. | ❑ | | ❑ | | ❑ | ❑ | ❑ |

Explanation: the next part of the questionnaire evaluates the need for guidance to exercise. Please answer the following questions.

| A) Are you satisfied with your current physical activities? | ❑ Yes❑ No, I would like to exercise more.❑ No, I would like to exercise less. | | |
| --- | --- | --- | --- |
| B) Do you need guidance/stimulation to change your exercise behaviour? | ❑ Yes 🡪 continue with question C.❑ No 🡪 this is the end of the questionnaire. | | |
| C) How often would you like to have guidance for exercising? | ❑ Weekly | ❑ Monthly | ❑ When I need it |
| D) Which form of guidance do you prefer?  *Choose your column of preference and then choose from this column the option which appeals the most to you.*  ***For this question it is possible to check multiple options. For example, you can prefer a therapist with specific skills in combination with a digital app on your smartphone or tablet.*** | ❑ *Individually:* | ❑ *In groups:* | ❑ *Digital* : |
|  | ❑ Physical therapist with specific skills, even if it is located outside my hometown. | ❑ At a regular gym, close to my hometown. | ❑ Using an application on my smartphone or tabloid. |
|  | ❑ Physical therapist, close to my hometown. | ❑ Together with systemic sclerosis patients, even if it is located outside my hometown. | ❑ Using email on tabloid, notebook or computer. |
|  | ❑ Personal trainer, for example connected with your gym, close to my hometown. | ❑ Together with patients with different (rheumatoid) diseases, even if it is located outside my hometown. | ❑ Using an (interactive) website on tabloid, notebook or computer. |
|  | ❑ Something else, that is: ________________ | ❑ Something else, that is: ________________ | ❑Something else, that is: _____________ |

Table 1 shows the complete data of each individual statement regarding physical activity promotion and guidance related to exercise. In total 26 topics regarding needs, preferences and perceptions on physical activity were assessed. Due to considerable overlap between part of the statements, data were summarized according to the subject addressed (see table 4 of the complete manuscript).

| **Table 1 “Complete data of each individual statement regarding physical activity promotion and guidance related to exercise”** | | |
| --- | --- | --- |
| Statements | Agreed or highly agreed, N (%) | Missing, N (%) |
| I (patient with systemic sclerosis) need more information about: |  |  |
| - Physical activity and sports | 34 (58) | 3 (5) |
| - Physical therapy | 31 (53) | 3 (5) |
| - Physical therapists with specific knowledge and skills regarding the management systemic sclerosis | 33 (56) | 3 (5) |
|  |  |  |
| Patients with systemic sclerosis |  |  |
| - Have their own responsibility to be sufficiently physically active | 54 (92) | 0 |
| - Rely on advice of their rheumatologists and general practitioners regarding PA and exercise | 17 (29) | 1 (2) |
| - *Can only engage in exercise or sports if they are accompanied* | 8 (14) | 1 (2) |
| - *Should get more help with making a choice for exercise or sports* | 20 (34) | 0 |
|  |  |  |
| The type of PA (including exercise and sports) I would prefer |  |  |
| - *Is not available in my neighbourhood* | 4 (7) | 3 (5) |
| - Is not appropriate for people with a disease such as systemic sclerosis | 10 (17) | 5 (9) |
|  |  |  |
| After being physically active I get a lot of pain. | 18 (31) | 3 (5) |
|  |  |  |
| I have the feeling that I |  |  |
| - Damage my joints too much by engaging in exercise and sports | 5 (9) | 2 (3) |
| - Damage my lungs too much by engaging in exercise and sports | 3 (5) | 3 (5) |
| - Damage my heart too much by engaging in exercise and sports | 3 (5) | 3 (5) |
| - Damage my skin too much by engaging in exercise and sports | 3 (5) | 2 (3) |
|  |  |  |
| *When participating with organized exercise activities for healthy persons I have the feeling of being watched by the other people because I have systemic sclerosis.* | 4 (7) | 2 (3) |
|  |  |  |
| In generally, I do not |  |  |
| - Have enough energy to engage in exercise and sports | 16 (27) | 0 |
| - Have enough time to engage in exercise and sports | 5 (9) | 1 (2) |
| - Want to engage in exercise and sports | 10 (17) | 1 (2) |
|  |  |  |
| *In my neighbourhood there is no professional support to* engage in exercise and sports | 4 (7) | 2 (3) |
| *I am not well enough informed about which possibilities for PA are available for systemic sclerosis patients.* | 16 (27) | 2 (3) |
| Sufficient levels of physical activity have a beneficial effect on the health status of persons with systemic sclerosis. | 48 (81) | 0 |
| *Daily activities, such as walking, for patients with systemic sclerosis could be improved by a better condition and more muscle strength.* | 44 (75) | 0 |
| I am satisfied with my current physical activities.* | 37 (63) | 3 (5) |
| I need guidance to exercise.*   - I would like to have guidance once a week.** - I would like to have guidance when I need it.** - I would like to have guidance individually.** - I would like to have guidance in groups.** | 13 (22)  8/13 (62)  2/13 (15)  9/13 (69)  6/13 (46) | 3 (5) |
