# Supplementary material for: Physical activity in patients with systemic sclerosis
Source: Rheumatol Int. 2017 Nov 18;38(3):443–53. doi: 10.1007/s00296-017-3879-y (PMC5847038; doi:10.1007/s00296-017-3879-y)
Supplement: Supplementary file 3 — Supplementary material 3 (DOC 82 KB) [file 296_2017_3879_MOESM3_ESM.doc]

**Physical Activity in Patients with Systemic Sclerosis**

SIE Liem, BSc^1^,

JMTA Meessen, MSc^2^,

R Wolterbeek, MSc^3^,

N Ajmone Marsan MD, PhD^4^,

MK Ninaber, MD, PhD^5^,

Prof. Dr. TPM Vliet Vlieland^2^,

JK de Vries-Bouwstra, MD, PhD^1^

^1^Department of Rheumatology, Leiden University Medical Centre, the Netherlands

^2^Department of Orthopedics, Leiden University Medical Centre, the Netherlands

^3^Department of Medical Statistics, Leiden University Medical Centre, the Netherlands

^4^Department of Cardiology, Leiden University Medical Centre, the Netherlands

^5^Department of Pulmonology, Leiden University Medical Centre, the Netherlands

**Corresponding author:**

J.K. de Vries-Bouwstra

Leiden University Medical Center, Department of Rheumatology, C1-51

PO Box 9600, 2300 RC Leiden, The Netherlands

Tel. [+31 71 526 3598](tel:+31 71 526 3598)

Fax.[+31 71 526 6752](tel:+31 71 526 6752)

E-mail [j.k.de_vries-bouwstra@lumc.nl](mailto:j.k.de_vries-bouwstra@lumc.nl)

**Appendix 3 “Comparison between included systemic sclerosis patients and the cohort”**

Table 1 shows the comparison between the included systemic sclerosis patients who completed the questionnaires and the cohort. The included SSc patients were significantly older than their cohort (65 years vs. 54 years, *P<0.001)*.

| **Table 1. Characteristics of systemic sclerosis patients (N=303)** | |  |  |
| --- | --- | --- | --- |
|  |  |  |  |
| **Sociodemographic characteristics** | Included SSc patients (N=59) | Cohort (N=244) | P-value |
| Age, years, median (interquartile range) | 65 (55-70) | 54 (45 – 65) | *<0.001* |
| Female, N (%) | 52 (88%) | 195 (81%) | 0.211 |
| Body Mass Index, kg/m^2^, mean (SD)* | 25 (4) | 25 (4) | 0.695 |
|  | |  |  |
| **Disease characteristics** | |  |  |
| Type of systemic sclerosis, *Diffuse*, N (%) | 15 (25%) | 60 (25%) | 0.768 |
| Duration of Raynaud’s phenomenon, years, median (interquartile range) | 12 (6-22) | 13 (6 -21) | 0.971 |
| Duration of non-Raynaud’s phenomenon, years, median (interquartile range) | 6 (3 – 16) | 7 (4 - 13) | 0.570 |
| Disease duration, years, median (interquartile range) | 4 (2-15) | 5 (2 - 12) | 0.574 |
| Modified Rodnan Skin Score, median (interquartile range) | 3 (0-6) | 4 (0 – 6) | 0.805 |
| Proximal muscular weakness or synovitis, N (%) | 6 (10%) | 32 (14%) | 0.473 |
| Gastrointestinal involvement, N (%)^ | 41 (76%) | 154 (66%) | 0.152 |
| Anti-Scl-70 antibodies, N (%) | 9 (15%) | 60 (25%) | 0.237 |
| Anti-centromere antibodies, N (%) | 30 (51%) | 77 (32%) | 0.006 |
| RNA polymerase III antibodies, N (%) | 3 (5%) | 14 (6%) | 0.868 |
| Interstitial lung disease according HRCT, N (%) | 26 (45%) | 129 (58%) | 0.082 |
| Pulmonary arterial hypertension, N (%) | 6 (11%) | 13 (6%) | 0.162 |
| Decreased ejection fraction, N (%) | 7 (13%) | 16 (11%) | 0.738 |
| Arrhythmia, N (%) | 24 (46%) | 89 (42%) | 0.586 |
| ***DLCO: diffuse capacity for carbon monoxide**  ^**Gastrointestinal involvement was defined as the presence of one of the following symptoms: reflux, early satiety, vomiting, diarrhoea, intestinal distension, constipation, faecal incontinence, parenteral nutrition or dysphagia.** | | | |
